# The Polysulfide‐Cathode Binding Energy Landscape for Lithium Sulfide Growth in Lithium‐Sulfur Batteries

**DOI:** 10.1002/advs.202206057

**Published:** 2023-03-01

**Authors:** Kiwon Kim, Jaehyun Kim, Jun Hyuk Moon

**Affiliations:** ^1^ Department of Chemical and Biomolecular Engineering Institute of Emergent Materials Sogang University Baekbeom‐ro 35, Mapo‐gu Seoul 04107 Republic of Korea

**Keywords:** DFT calculations, LiPS adsorption, lithium sulfide growth, lithium sulfur batteries, ternary oxide

## Abstract

A cathode substrate with strong adsorption of lithium polysulfides (LiPSs) has been preferred for lithium‐sulfur (Li‐S) batteries. However, the recent finding that controlled growth of lithium sulfides (Li_2_S) during discharge is crucial for S utilization stimulates improvement of this preference. Here, the Li_2_S growth and cell capacity in the LiPS binding energy landscape of cathode substrates are investigated. Specifically, Co‐based ternary oxides are employed to obtain binding energies in the range of 4.0–7.4 eV. Of these substrates, only the MnCo_2_O_4_ substrate with moderate LiPS affinity exhibits 3D Li_2_S growth. The MnCo_2_O_4_ cells achieve high sulfur utilization up to 84% at 0.2 C and excellent performance even under high sulfur loading/lean electrolyte conditions. In contrast, weak affinity substrates such as ZnCo_2_O_4_ and strong affinity substrates such as NiCo_2_O_4_ and CuCo_2_O_4_ exhibit low discharge capacity with 2D Li_2_S growth. For optimal LiPS affinity driving 3D growth, a balance between promoting LiPS adsorption and diffusion limitation in the LiPS adsorption layer is suggested.

## Graphical abstract

Lithium sulfide (Li_2_S) growth and cell performance are analyzed in the lithium polysulfide (LiPS) binding energy landscape of the cathode substrate. Among Co‐based ternary oxides, only the MnCo_2_O_4_ substrate with moderate LiPS affinity exhibits 3D Li_2_S growth and also achieves the highest cell performance.

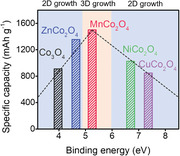

## 1 Introduction

Lithium‐sulfur (Li‐S) batteries are promising next‐generation energy storage devices with high theoretical and gravimetric energy densities of 1675 mAh g^−1^ and 2510 Wh kg^−1^, respectively.^[^ ^1^, ^2^, ^3^, ^4^ ^]^ Li‐S batteries employ a cathode that converts sulfur. Nevertheless, the currently achieved sulfur utilization at the cathode is unsatisfactory.^[^ ^5^ ^]^ A fundamental issue for regarding low sulfur utilization is that the lithium polysulfide (LiPS) intermediate produced during the charge/discharge process dissolves in the electrolyte with high solubility.^[^ ^6^ ^]^ The dissolved LiPSs cause permanent loss of sulfur through the so‐called “shuttle effect.”^[^ ^6^, ^7^ ^]^ For this reason, a cathode with a strong affinity for LiPSs has been preferred.^[^ ^8^ ^]^ Various oxides (e.g., TiO_2_, MnO_2_, V_2_O_5_, NiCo_2_O_4_, NiMoO_4_) have been widely introduced due to their chemical affinity for LiPSs.^[^ ^9^, ^10^, ^11^, ^12^, ^13^ ^]^ Various advanced substrates with strong LiPS binding energies (e.g., TiN, VN, Co_4_N, MoS_2_, CoS_2_) have also been applied.^[^ ^14^, ^15^, ^16^, ^17^ ^]^

However, recent studies have revealed that the growth morphology of lithium sulfide (Li_2_S) at the cathode, which is produced by the reduction of LiPSs during discharge, is crucial for sulfur utilization.^[^ ^18^, ^19^ ^]^ Li_2_S growth has been classified as 3D or 2D diffusion‐based growth according to classical electrodeposition theory.^[^ ^19^, ^20^, ^21^ ^]^ 2D growth conformally covers the substrate, and the electrically insulating Li_2_S passivates the substrate, preventing continuous sulfur conversion. In contrast, 3D growth favors complete sulfur utilization with the formation of particulate Li_2_S with less substrate contact.^[^ ^22^, ^23^ ^]^ The high sulfur utilization provided by 3D growth was first confirmed via control of the electrolyte donicity. Liu and coworkers, in their pioneering work, achieved 3D growth by controlling the electrolyte donicity using a mixture of 1,3‐dioxolane (DOL), 1,2‐dimethoxyethane (DME), and dimethyl sulfoxide (DMSO); they achieved near‐complete sulfur utilization under low C‐rate conditions.^[^ ^22^ ^]^ However, the high donicity electrolyte may delay the conversion of LiPSs due to strong solvation or adversely affect Li anode and other components due to strong reactivity.^[^ ^24^, ^25^ ^]^

These results suggest that a constraint should be added to the preference for strong LiPS adsorption at the cathode; the LiPS affinity that induces 3D Li_2_S growth should be explored. However, as the scope of many studies has been to introduce advanced cathodes, the effect of the cathode LiPS affinity on Li_2_S growth has not been elucidated. A few results comparing materials with different LiPS affinities have been presented, but there is an inconsistency between these results.^[^ ^16^, ^26^, ^27^ ^]^ Cao and co‐workers achieved 3D growth and high cell performance on strong LiPS adsorbent MoO/MoC in comparison of MoO_2_ and MoO/MoC substrates.^[^ ^26^ ^]^ Conversely, Qian and coworkers obtained 2D deposition on Co_4_N with the strongest binding energy among CoP, Co_4_N and CoS_2_.^[^ ^27^ ^]^ Therefore, exploring Li_2_S growth with respect to the LiPS affinity remains a challenge for practical Li‐S cells.

In this study, we present the LiPS adsorption energy landscape for the Li_2_S growth morphology using a Co‐based ternary oxide system. Partial substitution of cations in oxides changes the *d*‐band orbitals, resulting in systematic changes in the chemical affinity. Previously, ternary oxides have been applied to screen for oxygen evolution catalysts.^[^ ^18^, ^19^, ^28^ ^]^ In particular, the Co‐based ternary oxide allows many derivatives to tune the electronic structure over a wide range and has also been rarely applied to Li‐S batteries.^[^ ^20^, ^21^ ^]^ Here, we obtain LiPS binding energies in the range of 4.0–7.4 eV by partially replacing the cations in Co_3_O_4_ with Mn, Ni, Zn, and Cu. In the potentiostatic conversion of LiPSs to Li_2_S on these substrates, the MnCo_2_O_4_‐containing cathode with neither strong nor weak affinity exhibits 3D Li_2_S growth. As the LiPS affinity increases or decreases compared to that of MnCo_2_O_4_, the Li_2_S growth shifts to the 2D type. We confirm that the Li_2_S morphology determines the discharge capacity of the cell. The MnCo_2_O_4_‐based cathode delivers excellent cell capacity. The cells containing substrates with lower or higher affinity than MnCo_2_O_4_ exhibit lower cell performance. In particular, the MnCo_2_O_4_ cell achieves an initial capacity of 1398 mAh g^−1^ at 0.2 C, corresponding to a sulfur utilization of 84% and a high capacity of 1006 mAh g^−1^ even under high sulfur loading conditions (6 mg cm^−2^ and E/S 4.4). We suggest that the optimal affinity leading to 3D growth is determined by the balance of increased sulfur conversion induced by LiPS affinity and reduced conversion induced by diffusion limitation in the LiPS adsorbed layer.

## 2 Results and Discussion

### 2.1 Preparation of Co_3_O_4_‐Based Ternary Oxides

**Figure 1:**
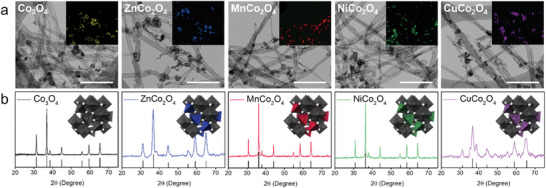
a) Transmission electron microscopy (TEM) images and b) XRD spectra of Co_3_O_4_‐, ZnCo_2_O_4_‐, MnCo_2_O_4_‐, NiCo_2_O_4_‐, and CuCo_2_O_4_‐coated CNT films. The inset image shows the mapping of the corresponding elements in the TEM image. The JCPDS reference peaks for Co_3_O_4_, ZnCo_2_O_4_, MnCo_2_O_4_, NiCo_2_O_4,_ and CuCo_2_O_4_ correspond to 23–1237, 23–1390, 20–0781, 25–0270, and 42–1467, respectively.

Co_3_O_4_‐based ternary oxides are prepared by reductive precipitation of Co and other cation salts (Experimental Section and Figure S1, Supporting Information).^[^ ^18^ ^]^ Co_3_O_4_ nanoparticles are synthesized using the same procedure, but only with Co cation salts. Oxide nanoparticle‐coated‐carbon nanotube (CNT) composite films are obtained by reaction in a solution in which CNTs are dispersed (Figure S2, Supporting Information). The reaction time is controlled so that the nanoparticles have similar sizes and the nanoparticle‐coated CNT films have similar Brunauer–Emmett–Teller (BET) surface areas. The ternary oxides and Co_3_O_4_ nanoparticles are uniformly coated on the CNT films with a size of 20–40 nm, as shown in **Figure** **1**a (Figure S3, Supporting Information). The BET surface areas of these composite samples are similar, in the range of 13–15 m^2^ g^−1^ (Figure S4, Supporting Information). The nanoparticle content in these composite samples is 18–20 wt%, showing only a small variation (Figures S5 and S6, Supporting Information).

The X‐ray diffraction (XRD) spectrum of each ternary oxide sample shows diffraction peaks consistent with the JCPDS reference of each material (Figure 1b). No secondary peaks other than these diffraction peaks are observed, indicating the absence of phase‐separated oxides. All ternary oxides exhibit the spinel structure (the *Fd* $\bar{3}$ *m* space group); the two‐cation oxides form a tetrahedral structure and an octahedral structure.^[^ ^18^, ^29^ ^]^ The strongest peak corresponds to the (311) plane, characteristic of the spinel structure.^[^ ^30^, ^31^ ^]^ We calculate the lattice parameters using the Bragg equation with the (311) peak.^[^ ^32^ ^]^ The lattice parameters for MnCo_2_O_4_, ZnCo_2_O_4_, NiCo_2_O_4_, and CuCo_2_O_4_ are 0.24, 0.25, 0.24 and 0.24 nm, respectively, consistent with the results in the literature (Figure S7, Supporting Information).

### 2.2 Affinity for Lithium Polysulfides

**Figure 2:**
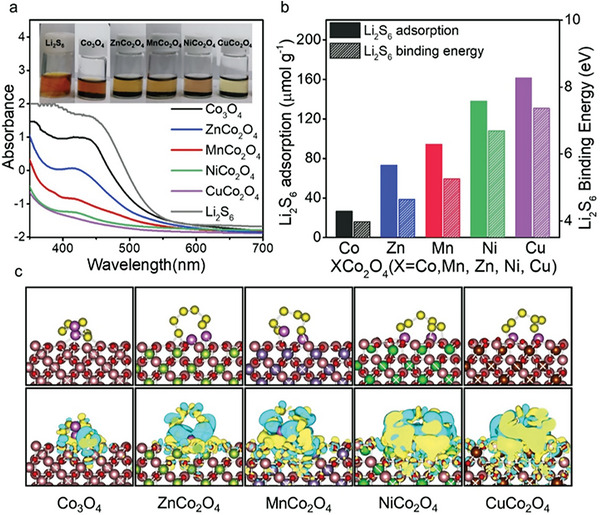
a) UV–vis absorption spectrum of the electrolyte after adsorption in 2 mm Li_2_S_6_ electrolyte containing each ternary oxide or Co_3_O_4_. The inset images show a digital camera image of each electrolyte. b) Adsorption amount of Li_2_S_6_ from the absorption spectrum, and BE of Li_2_S_6_ from the DFT calculation. c) Optimized atomic configuration and charge density difference of Li_2_S_6_ adsorbed on each substrate. The (400) plane of these oxides is defined as the surface.^[^ ^35^, ^36^ ^]^ The isosurface level is set at 0.003 e Å^−3^.

We analyze the Li_2_S_6_ adsorption capacity for MnCo_2_O_4_, CuCo_2_O_4_, NiCo_2_O_4_, ZnCo_2_O_4_, and Co_3_O_4_. The electrolyte solution containing these oxide nanoparticles is decolorized, revealing the adsorption of Li_2_S_6_ by the nanoparticles (**Figure** **2**a); the Li_2_S_6_ adsorption capacity decreases in the order of CuCo_2_O_4_, NiCo_2_O_4_, MnCo_2_O_4_, ZnCo_2_O_4_, and Co_3_O_4_. We quantify the adsorption capacity of each oxide particle using the peak intensity at 420 nm corresponding to S_6_^2−^ absorption in the UV–vis absorption spectrum. The adsorption capacities for CuCo_2_O_4_, MnCo_2_O_4_, NiCo_2_O_4_, ZnCo_2_O_4_, and Co_3_O_4_ are 146, 138, 104, 73, and 27 µmol g^−1^, respectively (Figure 2b and Note S1, Supporting Information).^[^ ^33^, ^34^ ^]^

We perform density functional theory (DFT) calculations to obtain the Li_2_S_6_ binding energies (BEs) of the ternary oxides and Co_3_O_4_. The optimal atomic configuration of Li_2_S_6_ adsorbed on MnCo_2_O_4_, ZnCo_2_O_4_, NiCo_2_O_4_, CuCo_2_O_4,_ or Co_3_O_4_ is displayed in Figure 2c. The calculated BEs on each substrate are plotted in Figure 2b, where the order of the BEs is consistent with that of the experimental adsorption capacities. The absolute BEs of these oxides range from 4.0 to 7.4 eV. Note that this range is wide enough given that the BEs of LiPS adsorbents in prior studies are in the range of ≈2–9 eV (Note S2, Supporting Information).

We analyze the charge density difference in the binding between these substrates and Li_2_S_6_ (Figure 2c). The electron localization between S_6_^2−^ and Co_3_O_4_ is relatively weak in the case of Co_3_O_4_, and increases in the order of ZnCo_2_O_4_, MnCo_2_O_4_, NiCo_2_O_4_, and CuCo_2_O_4_. An increase in the charge localization indicates an increase in the covalent bonding nature of the cation element and Li.^[^ ^34^, ^37^ ^]^ The difference in the BEs for these oxide substrates is determined by their covalent properties.

### 2.3 Li_2_S Growth on the Cathode Substrate

**Figure 3:**
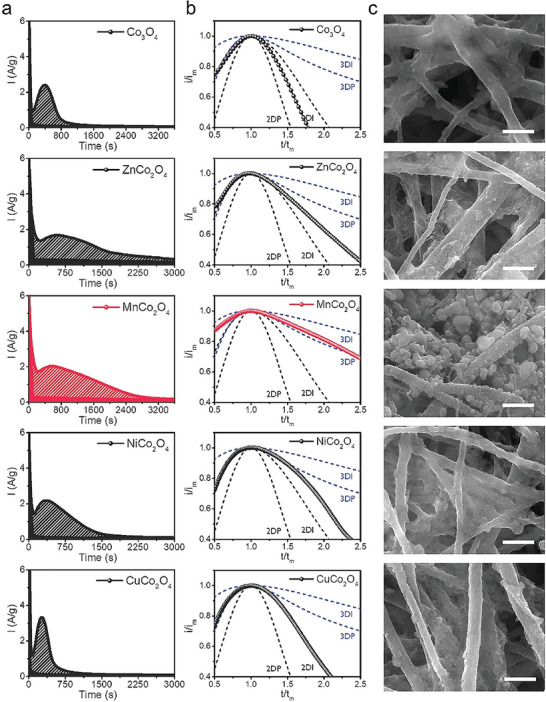
a) Potentiostatic discharge *i–t* curve, b) dimensionless *i–t* curve, and c) SEM image of the Li_2_S‐grown substrate for CuCo_2_O_4_‐, MnCo_2_O_4_‐, NiCo_2_O_4_‐, ZnCo_2_O_4_‐, and Co_3_O_4_‐coated CNT cathode cells.

The precipitation of Li_2_S via reduction of liquid‐phase LiPSs on the ternary oxide and Co_3_O_4_ substrates is investigated by chronoamperometry. The potentiostatic discharge *i–t* curves of each substrate electrode are presented in **Figure** **3**a. The shaded area under the discharge curve corresponds to the precipitation capacity. We obtain a dimensionless profile using the maximum current (*i*_m_) and a specific time (*t*_m_) (Figure 3b and Note S3, Supporting Information). The dimensionless profile is employed to distinguish the nucleation‐growth type of Li_2_S.^[^ ^23^, ^38^ ^]^ The profiles for 2D and 3D electrodeposition shown in Figure 3b are obtained using the Bewick, Fleischman, and Thirsk (BFT) and Scharifker–Hills (SH) models, respectively.^[^ ^23^, ^34^ ^]^ Specifically, 2D and 3D electrodeposition are based on growth via surface electrochemical reactions and radial 3D mass transfer‐controlled growth, respectively. For each type of growth, instantaneous (I) or progressive (P) nucleation is considered.^[^ ^39^, ^40^ ^]^ We also present ex situ scanning electron microscopy (SEM) images of Li_2_S on each substrate in Figure 3c.

The MnCo_2_O_4_ cathode, which has a moderate binding affinity for LiPSs, achieves the highest precipitation capacity (627 mAh g^−1^) with a much longer discharge time than the other substrate cathodes. The dimensionless *i–t* profile for the MnCo_2_O_4_ substrate corresponds to 3D growth with progressive nucleation. The ex situ SEM image confirms the growth of particulate Li_2_S on MnCo_2_O_4_/CNT (Figure S8, Supporting Information).

When the binding affinity of the nanoparticle substrate is weaker or stronger than that of MnCo_2_O_4_, the precipitation capacity decreases, and the growth mode deviates from the 3D type (see Figure 3 and Figure S8, Supporting Information). ZnCo_2_O_4_, with weaker affinity than MnCo_2_O_4_, exhibits a low capacity of 574 mAh g^−1^ and a 2D growth profile. The substrate with the weakest affinity, Co_3_O_4_, shows full 2D growth with the lowest capacity of 433 mAh g^−1^. The NiCo_2_O_4_ substrate, which has a stronger binding affinity than MnCo_2_O_4_, exhibits a profile close to that of 2D growth. Growth on the substrate with the strongest affinity, CuCo_2_O_4_, corresponds to full 2D growth (Figure 3). The capacities for the NiCo_2_O_4_ and CuCo_2_O_4_ substrates are relatively low at 512 and 358 mAh g^−1^, respectively.

### 2.4 Comparison of Cell Performance

**Figure 4:**
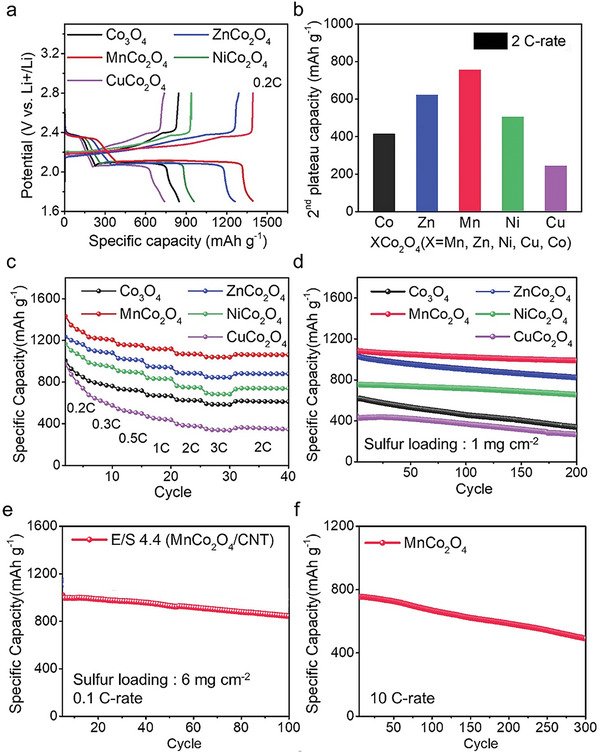
a) Charge/discharge profile, b) 2nd plateau capacity, c) rate performance, and d) cycle performance at low sulfur loading condition (current density: 3.35 mA cm^−2^) for CuCo_2_O_4_/CNT, MnCo_2_O_4_/CNT, NiCo_2_O_4_/CNT, ZnCo_2_O_4_/CNT, and Co_3_O_4_/CNT cells. e) Specific capacity at high sulfur loading/lean electrolyte and f) at 10 C‐rate for the MnCo_2_O_4_/CNT cell.

Li‐S batteries, each including a cathode substrate of CuCo_2_O_4_/CNT, MnCo_2_O_4_/CNT, NiCo_2_O_4_/CNT, ZnCo_2_O_4_/CNT, or Co_3_O_4_/CNT are prepared. The discharge/charge profiles at 0.2 and 2 C rates for these cells are shown in **Figure** **4**a and Figure S9 (Supporting Information), respectively. The discharge capacities of the MnCo_2_O_4_/CNT, ZnCo_2_O_4_/CNT, NiCo_2_O_4_/CNT, CuCo_2_O_4_/CNT, and Co_3_O_4_/CNT cells at 0.2 C are 1398, 1261, 955, 847 and 789 mAh g^−1^, respectively. We also compare the capacities at the 2nd discharge plateau (2.08–2.02 V vs Li^+^/Li, Li_2_S_4_ to Li_2_S conversion) as shown in Figure 4b; the 2nd plateau corresponds to the reduction of LiPSs to Li_2_S. The 2nd plateau capacity is highest for MnCo_2_O_4_/CNT, and then decreases in the order of ZnCo_2_O_4_/CNT, NiCo_2_O_4_/CNT, Co3CuCo_2_O_4_/CNT, and Co_3_O_4_/CNT. Thus, the discharge capacity is determined by the capacity in the Li_2_S growth. This result confirms that the MnCo_2_O_4_ substrate exhibiting 3D Li_2_S growth delivers the highest capacity. The more the substrate exhibits growth deviating from 3D growth, the greater the decrease in the discharge capacity. These results are consistent with the prior results that obtained high sulfur utilization in 3D growth.^[^ ^23^, ^38^ ^]^

The discharge capacity of each substrate cell for a 15‐fold increase in the C‐rate from 0.2 to 3 C is shown in Figure 4c (Figure S10, Supporting Information). The MnCo_2_O_4_/CNT cells retain 85% of their initial capacity at this C‐rate increase. The Co_3_O_4_ and ZnCo_2_O_4_ cells achieve retention rates of 73% and 76%, respectively. The NiCo_2_O_4_ and CuCo_2_O_4_ cells achieve retention rates of 70% and 52%, respectively. The MnCo_2_O_4_/CNT electrode with 3D Li_2_S growth exhibits the highest capacity retention. Previously, it has been observed that at high C‐rates, the nucleation of Li_2_S occurs more instantaneously, accelerating surface passivation.^[^ ^26^, ^41^ ^]^ Thus, alleviation of surface passivation by 3D growth on the MnCo_2_O_4_/CNT leads to superior capacity retention at increasing C‐rate; In contrast, the substrates with 2D growth have significantly lower capacity retention with faster passivation at increasing C‐rate.

The charge/discharge cycle stability for the MnCo_2_O_4_/CNT, ZnCo_2_O_4_/CNT, NiCo_2_O_4_/CNT, CuCo_2_O_4_/CNT, and Co_3_O_4_/CNT cells is shown in Figure 4d. The MnCo_2_O_4_ cell exhibits the highest cycle stability at 200 cycles (decay rate per cycle: 0.041%) (see Figure S10, Supporting Information). The ZnCo_2_O_4_ and Co_3_O_4_ cells show capacity losses of 0.098% and 0.265% with cycling, respectively; the weak‐LiPS‐affinity substrate is insufficient in inhibiting the shuttling of LiPSs during cycling.^[^ ^42^, ^43^ ^]^ Interestingly, strong‐LiPS‐affinity substrates also exhibit poor cycle stability; the capacity losses per cycle for the NiCo_2_O_4_/CNT and CuCo_2_O_4_/CNT cells are 0.045% and 0.128%, respectively. These results suggest that not only the adsorption of LiPS but also the conversion to Li_2_S with suppressed passivation are crucial for capacity retention even in long‐term charge–discharge cycles.^[^ ^22^, ^30^ ^]^

Note that the performance of the moderate adsorbent MnCo_2_O_4_/CNT cells is excellent. The sulfur utilization reaches 84% at 0.2 C. The discharge capacity of 1184 mAh g^−1^ at 2 C is higher than the capacities achieved in recent studies employing advanced cathode substrates (see Note S2, Supporting Information). The initial discharge capacity under high sulfur loading conditions (1143 mAh g^−1^ at 0.1 C, 6 mg cm^−2^ and E/S 4.4) is also superior to the results in the literature highlighting high sulfur loading and lean electrolyte conditions: NiMoO_4_ (900 mAh g^−1^ at 0.1 C, 5.5 mg cm^−2^ and E/S 4), Ta_2_O_5−x_ (892 mAh g^−1^ at 0.2 C, 5.6 mg cm^−2^ and E/S 3.6).^[^ ^11^, ^31^, ^39^, ^43^ ^]^ In addition, the MnCo_2_O_4_/CNT cell achieves initial capacity of 760 mAh g^−1^ and capacity of 491 mAh g^−1^ even after 300 cycles under ultra‐fast cycling at 10 C‐rate. This achievement is about 70% higher discharge capacity than a Li‐ion battery with only 100 s of charging.

Briefly, these measurements of various cell performances reveal that the LiPS affinity of MnCo_2_O_4_ is close to the global optimum achieving high cell performance.

### 2.5 Growth Mechanism of Li_2_S

We observe the evolution of Li_2_S growth from 2D to 3D and back to 2D with increasing LiPS affinity. The MnCo_2_O_4_ substrate with the “Goldilocks” affinity, neither too strong nor too weak, exhibits 3D growth, accompanied by the highest cell performance. The remaining question is why there is an optimal affinity for 3D growth in the LiPS BE landscape.

**Figure 5:**
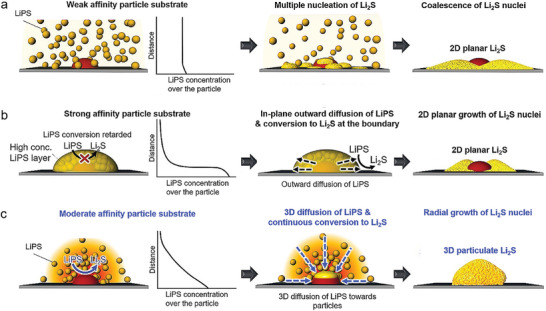
Comparison of the mechanisms for the precipitation growth of Li_2_S from liquid‐phase LiPSs on substrates with a) weak, b) strong, and c) moderate affinity particles. Weak affinity particle substrate: the Li_2_S nucleation occurs throughout the substrate, and the coalescence of these nuclei forms 2D Li_2_S (a, top panel). Strong affinity particle substrate: the diffusion of LiPS from the strong LiPS layer around the particle to the periphery and the subsequent LiPS‐to‐Li_2_S conversion form 2D Li_2_S (b, middle panel). Moderate affinity particle substrate: The diffusion toward the particle and the conversion to Li_2_S proceed sequentially, where the 3D diffusion of LiPS results in 3D isotropic Li_2_S growth (c, bottom panel).

The growth of Li_2_S on weak‐LiPS‐affinity nanoparticle/CNT substrates (Co_3_O_4_/CNT and ZnCo_2_O_4_/CNT) may be similar to the growth on bare carbon substrates. This is because the nanoparticles with a LiPS affinity that is not significantly strong compared to carbon are not capable of selective and rapid formation of Li_2_S nuclei. 2D growth in the dimensionless *i–t* curve has been confirmed on carbon substrates such as CNTs and carbon fibers.^[^ ^23^, ^24^, ^40^ ^]^ We suggest instantaneous and uniform Li_2_S nucleation on nanoparticles and carbon surfaces, as depicted in **Figure** **5**a. The coalescence following the growth of these nuclei forms the 2D Li_2_S layer.

Interestingly, 2D growth is observed even with strong‐LiPS‐affinity nanoparticle/CNT substrates (CuCo_2_O_4_/CNT and NiCo_2_O_4_/CNT). We suggest that the formation of a highly viscous layer induced by excessive adsorption of LiPSs on these particle substrates makes the LiPS conversion diffusion‐limited (Figure 5b). To confirm this, we analyze the series resistance according to the depth of discharge (DOD) by *operando* electrochemical impedance spectroscopy (EIS) (see Figure S11, Supporting Information).^[^ ^35^, ^36^, ^44^ ^]^ A DOD of 25–30% corresponds to the LiPS‐rich state of the electrolyte. The NiCo_2_O_4_ and CuCo_2_O_4_ substrate cells show surging resistance in this DOD region; in particular, the CuCo_2_O_4_ cell maintains high resistance even at a DOD above 30%, which demonstrates the formation of a high‐viscosity LiPS layer on these substrates. The diffusion limitation of LiPSs on these particles may cause 2D outward diffusion of LiPSs (Figure 5b). This surface 2D diffusion results in 2D Li_2_S growth.

The above scenario predicts the selective adsorption of LiPSs and the formation of 3D LiPS diffusion regions on the MnCo_2_O_4_ particle substrate. Specifically, in the moderate affinity MnCo_2_O_4_ particles, the formation of a gradual LiPS concentration gradient continues diffusion of LiPS toward the particle, and also does not exacerbate the conversion to Li_2_S (Figure 5c). As a result, the Li_2_S nuclei formed around the MnCo_2_O_4_ particles undergo continuous isotropic 3D growth with a 3D diffusion region, forming particulate Li_2_S (Figure 5c). We compare the Li_2_S_6_‐Li_2_S conversion kinetics for each substrate via symmetric electrode cyclic voltammetry (CV), confirming the fastest conversion for the MnCo_2_O_4_/CNT substrate (Note S4, Supporting Information); this confirms that sustained nucleation/growth proceeds around MnCo_2_O_4_ without slow nucleation (on weak adsorbent substrates) or diffusion‐limited 2D growth (on strong adsorbent substrates).

## 3 Conclusion

We employ Co_3_O_4_‐based ternary oxides to obtain substrates with various LiPS affinities. A CNT film coated with Co_3_O_4_ and ternary oxide nanoparticles is applied as a cathode substrate. The *i–t* profile and ex situ SEM images in potentiostatic discharge distinguish the growth type of Li_2_S at the cathode. The MnCo_2_O_4_ substrate with moderate LiPS affinity exhibits 3D Li_2_S growth. In contrast, weak‐affinity substrates (ZnCo_2_O_4_ and Co_3_O_4_) and strong‐affinity substrates (CuCo_2_O_4_ and NiCo_2_O_4_) compared to MnCo_2_O_4_ lead to 2D growth. We confirm that the growth type in these cathode cells is highly correlated with the cell performance. The MnCo_2_O_4_ substrate cathode cell achieves superior performance compared to the other substrate cells; excellent performance is achieved at a high C‐rate, over several charge/discharge cycles, and under high sulfur loading/lean electrolyte conditions. The MnCo_2_O_4_ cell is also superior to prior results. To account for the optimal LiPS affinity leading to 3D growth, we present a balance between promoting LiPS adsorption and diffusion limitation in the LiPS adsorption layer. The MnCo_2_O_4_ substrate promotes the adsorption of LiPSs but does not result in a viscous LiPS adsorption layer. This causes continuous 3D diffusion of LiPSs and consequently isotropic growth of particulate Li_2_S. The Goldilocks affinity cathode will be the redefined selection criterion for practical Li‐S batteries.

## 4 Experimental Section

### Preparation of Ternary Oxide Nanoparticles/CNTs

CNT film was prepared by the vacuum filtration method. The solution in which MWCNT (Aldrich) was dispersed using Triton X‐100 surfactant was vacuum filtered using a PVdF membrane filter. To prepare MnCo_2_O_4_ nanoparticles, first, a mixed solution of 0.1 m Mn(NO_3_)_2_•6H_2_O and 0.2 m Co(NO_3_)_2_•6H_2_O was prepared, and 0.5 m NaOH was added dropwise thereto to obtain hydroxide. A solution of this hydroxide dispersed in ethanol was coated on free‐standing CNTs and dried, followed by sintering at 400 °C for 2 h to produce a CNT film coated with MnCo_2_O_4_ nanoparticles. The preparation of ZnCo_2_O_4_, NiCo_2_O_4_, and CuCo_2_O_4_ particles and each coated CNT film was obtained by the same procedure; for ZnCo_2_O_4_, NiCo_2_O_4_, and CuCo_2_O_4_, salts of Zn(NO_3_)_2_•6H_2_O, Ni(NO_3_)_2_•6H_2_O, and Cu(NO_3_)_2_•3H_2_O were used, respectively.

### Adsorption Analysis

The Li_2_S_6_‐containing electrolyte solution was prepared by stirring lithium sulfide (Li_2_S, Alfa Aesar) and sulfur powder (S_8_, Alfa Aesar) at a molar ratio of 8:5 in DOL/DME (v/v% = 1:1) at 90 °C. This Li_2_S_6_ solution was diluted to a concentration of 0.2 mm using DOL/DME. The adsorption measurements were obtained by recording UV–vis spectra over time of a 0.2 mm Li_2_S_6_ solution containing 20 mg oxide.

### DFT Calculation

The DFT calculation of the adsorption energy of Li_2_S_6_ was carried out using the Quantum ESPRESSO package.^[^ ^45^ ^]^ The interaction between the core and valence electrons was described by the frozen‐core projector augmented wave (PAW) approach. The generalized gradient approximation (GGA) of the Perde–Burke–Ernzerhof (PBE) was applied as an exchange‐correlation functional. The van der Waals (vdW) interactions were applied by using DFT‐D3 of Grimme. A plane‐wave basis set with an energy cutoff of 450 eV was adopted to expand the electronic wavefunctions. For the calculation, Monkhorst–Pack meshes with 1 × 3 × 3 k‐point mesh were used to sample the two‐dimensional Brillouin zone, and a vacuum spacing of 20 Å was constructed in the (400) direction. Energy precision of 10^−5^ eV was employed, and atomic coordinates were relaxed until the maximum residual force was less than 0.02 eV Å^−1^. The binding energy (*E*_B_) between Li_2_S_6_ and metal oxide was calculated using the equation,^[^ ^46^ ^]^

(1) $$E_{B}=E\left({\mathrm{Li}}_{2}S_{6}+\mathrm{sub}.\right)-E\left({\mathrm{Li}}_{2}S_{6}\right)-E\left(\mathrm{sub}.\right)$$

where *E*(Li_2_S_6_+sub.), *E*(Li_2_S_6_), and *E*(sub.) denote the energy for the Li_2_S_6_‐adsorbed system, Li_2_S_6_, and substrate, respectively.

The charge density difference (∆*ρ*) was calculated using the equation,^[^ ^27^ ^]^

(2) $$\Delta \rho =\rho \left({\mathrm{Li}}_{2}S_{6}+\mathrm{sub}.\right)-\rho \left({\mathrm{Li}}_{2}S_{6}\right)-\rho \left(\mathrm{sub}.\right)$$

where *ρ*(Li_2_S_6_+sub), *ρ*(Li_2_S_6_), and *ρ*(sub) denote the charge density for the Li_2_S_6_‐adsorbed system, Li_2_S_6_, and substrate at atomic configuration optimized from the calculation of *E*_B_, respectively. The isosurface level was set at 0.003 e Å^−3^.

### Assembly of Li‐S Cells

The cathode for lithium‐sulfur batteries was obtained by loading sulfur on an oxide‐coated CNT cathode substrate by conventional melt diffusion. The sulfur loading per area was typically 1 mg cm^−2^. The content of S relative to the total cathode mass was controlled to be about 75%. The E/S value was set to 15. For high sulfur loadings and lean electrolytes, the areal loading and the E/S were controlled to be 6 mg cm^−2^ and 4.4–6.5, respectively. The cell was obtained by sulfur‐loaded oxide/CNT cathode, Li foil anode, and separator (Celgard 2400). The geometric sizes of the cathode electrode, separator, and anode electrode had a diameter of 12, 16, and 16 mm, respectively. The electrolyte was prepared by dissolving 1.2 m lithium bis(trifluoromethane) sulfonimide (LiTFSI, Sigma‐Aldrich) and 0.2 m lithium nitrate (LiNO_3_, Alfa Aesar) dissolved in 1,3‐dioxolane (DOL, Sigma‐Aldrich)/1,2‐dimethoxyethane (DME, Sigma‐Aldrich) (v/v% = 1:1).

### Electrochemical Measurements

All cell tests were performed using a Maccor 4300 Battery Test System. For the galvanostatic charge and discharge, the voltage range was controlled over 1.7–2.8 V (vs Li/Li^+^). The chronoamperometry was performed by applying a constant voltage of 2.05 V (vs Li/Li^+^) to a cell containing a Li_2_S_6_ catholyte. The cyclic voltammetry was performed the voltage range of 1.7–2.8 V (vs Li/Li^+^) at various scan rates from 0.2 to 0.5 mV s^−1^.

### Materials Characterization

The field‐emission scanning electron microscopy (FE‐SEM, JSM‐7100F) and the transmission electron microscopy (TEM, JEM‐3010, JEOL) were used to characterize microstructures. The X‐ray diffraction (XRD, Davinci D8 Advance diffractometer) was obtained with a scan rate 0.05° s^−1^ in scan ranges of 10^–^80°. The X‐ray photoelectron spectroscopy (XPS) was collected in a Leybold spectrometer with an Al K*α* monochromatic beam (1486.6 eV) (150 W input power, ESCALAB250 XPS system, Theta Probe XPS system). The Barrett–Emmett–Teller (BET) method (ASAP 2020, Micrometrics Inc.) was carried out to obtain the specific surface areas.

## Conflict of Interest

The authors declare no conflict of interest.

## Supporting information

Supporting Information

## Data Availability

The data that support the findings of this study are available from the corresponding author upon reasonable request.
