# Supplementary material for: The Polysulfide‐Cathode Binding Energy Landscape for Lithium Sulfide Growth in Lithium‐Sulfur Batteries
Source: Adv Sci (Weinh). 2023 Mar 1;10(12):2206057. doi: 10.1002/advs.202206057 (PMC10131804; doi:10.1002/advs.202206057)
Supplement: Supplementary file 1 — Supporting Information [file ADVS-10-2206057-s001.pdf]

ADVANCED SCIENCE
Open Access

# Supporting Information

for *Adv. Sci.*, DOI 10.1002/advs.202206057

The Polysulfide-Cathode Binding Energy Landscape for Lithium Sulfide Growth in Lithium-Sulfur Batteries

*Kiwon Kim, Jaehyun Kim and Jun Hyuk Moon**

# Supporting Information

**The polysulfide-cathode binding energy landscape for lithium sulfide growth in lithium-sulfur batteries**

*Kiwon Kim, Jaehyun Kim, and Jun Hyuk Moon**

K. Kim, J. Kim, Prof. J. H. Moon*
Department of Chemical and Biomolecular Engineering, Institute of Emergent Materials, Sogang University, Baekbeom-ro 35, Mapo-gu, Seoul, 04107, Republic of Korea

**Corresponding Author**
Jun Hyuk Moon, E-mail: junhyuk@sogang.ac.kr

**Supplementary note #1**

We analyze the chemical interaction between $Li_2S_6$ and ternary oxide nanoparticles by XPS. The shift of the S 2p peak exhibited by $Li_2S_6$ adsorbed on oxide nanoparticles is proportional to the oxide-LiPS interaction. The S 2p spectra for $MnCo_2O_4$, $ZnCo_2O_4$, $NiCo_2O_4$, $CuCo_2O_4$, and $Co_3O_4$ exhibit doublet peaks near 162 eV and 164 eV, respectively. Each peak corresponds to the terminal ($S_T^{-1}$) and bridging ($S_B^0$) configurations of the adsorbed sulfur species. The strong intensity peaks exhibited above 166 eV are those for polythionate and thiosulfate

We compare the positions of the peaks corresponding to $S_B^0$ and $S_T^{-1}$. The $S_B^0$ peaks for $MnCo_2O_4$, $ZnCo_2O_4$, $NiCo_2O_4$, $CuCo_2O_4$, and $Co_3O_4$ are located at 163.2 eV, 163.1 eV, 163.6, 163.1 eV, and 163.7 eV, respectively. The $S_T^{-1}$ peaks for $MnCo_2O_4$, $ZnCo_2O_4$, $NiCo_2O_4$, $CuCo_2O_4$ and $Co_3O_4$ are located at 161.7 eV, 161.6 eV, 162.0 eV, 161.5 eV and 161.8 eV, respectively. This result indicates that LiPS binding is weakened in the order of $CuCo_2O_4$, $NiCo_2O_4$, $MnCo_2O_4$, $ZnCo_2O_4$, and $Co_3O_4$. It has been previously reported that the positive shift of the peaks corresponding to $S_B^0$ and $S_T^{-1}$ is induced by high electron polarization by strong interaction between LiPS and oxide. This result exactly matches the order of binding energy by DFT calculation.

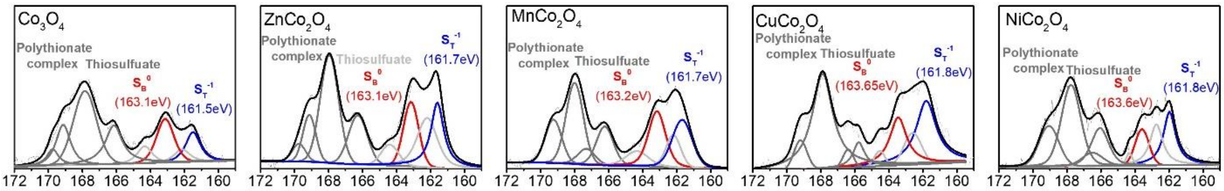


**Figure**. S 2p XPS spectra of $MnCo_2O_4$, $ZnCo_2O_4$, $NiCo_2O_4$, $CuCo_2O_4$, and $Co_3O_4$ adsorbed with $Li_2S_6$, respectively.

**Supplementary note #2**

The cathode substrates employed in the prior results exhibited binding energies ranging from -2 to -9 eV.

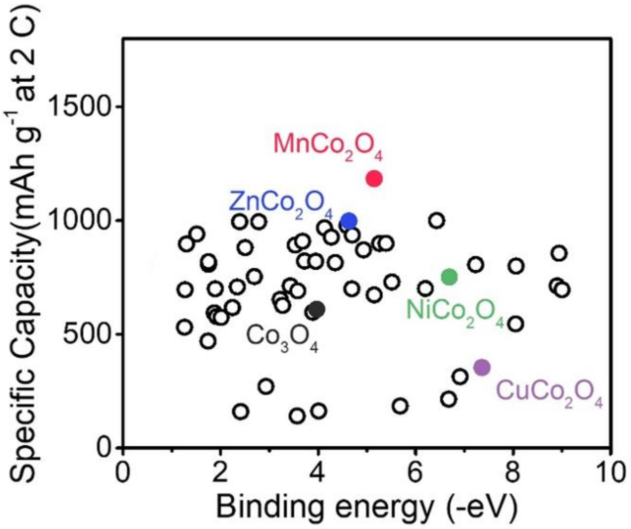


**Figure**. Mapping of the calculated LiPS binding energy versus discharge capacity at 2 C for the cathode substrates employed in the previous results. Our results are marked with a filled circle.

**Table**. LiPS adsorption substrate, binding energy and discharge capacity for the results in the plot.

| LiPS affinity substrate | Binding energy for $Li_2S_6$ (- eV) | Discharge capacity (mAh $g^{-1}$) |
|---|---|---|
| $MnCo_2O_4$ (Our) | 5.25 | 1184 |
| MnO | 1.75 | 808[4] |
| ZnSe | 1.74 | 470[5] |
| $Ni_2Co$ | 1.87 | 594[6] |
| CoP | 1.89 | 700[7] |
| NiO | 1.92 | 578[8] |
| $V_2O_3$ | 2.01 | 574[9] |
| $Fe_3O_4$ | 2.24 | 617[10] |
| $NiTe_2$ | 2.34 | 708[11] |
| $SnP_{0.94}$ | 2.41 | 160[12] |
| CoSe-ZnSe | 2.5 | 881[5] |
| NbC-Co | 2.69 | 754[13] |
| Co-$MoSe_2$ | 2.78 | 995[14] |
| $Co(OH)_2$ | 2.93 | 995[15] |
| $V_8C_7$ | 3.27 | 627[9] |
| $NiCo(OH)_2$ | 3.43 | 713[15] |

| | | |
|---|---|---|
| $Fe_9S_{10}/Fe_3O_4$ | 3.53 | 892[10] |
| $TiO_{2-x}$ | 3.57 | 141[16] |
| $NiMoO_4$ | 3.58 | 691[8] |
| CoP-Vp | 3.72 | 822[7] |
| C doped $TiO_{2-x}$ | 3.89 | 597[16] |
| Amorphous CoP | 3.95 | 821[17] |
| C doped $TiO_2$ | 4.01 | 163[16] |
| $FeSe_2$ | 4.27 | 927[18] |
| $NiTe_{2-x}$ | 4.35 | 815[11] |
| $Fe_3C$ | 4.59 | 978[19] |
| $TiB_2$ | 4.69 | 700[20] |
| P doped $Co_9S_8$ | 4.78 | 936[21] |
| P-$Co_9S_8$ | 4.93 | 871[21] |
| $Nb_2O_5$-NbC | 5.13 | 673[10] |
| P doepd $NiTe_{2-x}$ | 5.26 | 899[11] |
| $CoO_{1-x}$ | 6.2 | 701[22] |
| $Fe_2N$ | 6.43 | 1000[23] |
| Pt | 6.68 | 215[24] |
| $MgB_2$ ($Li_2S_4$) | 6.8 | 1000 (0.5C)[25] |
| CoO | 6.91 | 314[22] |
| PtNi | 8.05 | 545[24] |
| $Mo_2C$ | 8.06 | 800[26] |
| G-N-$Fe_3C$ | 8.9 | 713[27] |
| Fe/$C_2N$ | 8.94 | 856[28] |
| ZnSe | 9 | 696[29] |

**Supplementary note #3**

The current-time (*i*-*t*) curve obtained by chronoamperometry is compared with the classical electrochemical deposition model.[30-31] We utilize Bewick, Fleischman, and Thirsk (BFT) and Scharifker-Hills (SH) models to approximate the 2D and 3D growth, respectively.[30, 32] The 2D growth model describes the formation of a planar deposition layer by merging adjacent nuclei. In contrast, the 3D growth describes the growth by three-dimensional volumetric diffusion of precursors.[30-32]

The table below contains equations for the 2D and the 3D growth. We distinguish between instantaneous (I) and progressive (P) nucleation in each growth type. We introduce parameters to simplify the equation; the $i_m$ stands for peak current, and the $t_m$ corresponds to time at $I_m$.

| Type | Equation | $\boldsymbol{I_m t_m}$ or $\boldsymbol{I_m^2 t_m}$ | $\boldsymbol{t_m}$ |
|---|---|---|---|
| 2DI | $\frac{I}{I_m} = \frac{t}{t_m}\{\exp[-\frac{t^2 - t_m^2}{2t_m^2}]\}$ | $I_m t_m = \frac{nF\rho h}{M}\{\exp[-\frac{1}{2}]\}$ | $t_m = \sqrt{\frac{\rho^2}{2\pi M^2 N_0 k_g^2}}$ |
| 2DP | $\frac{I}{I_m} = \left(\frac{t}{t_m}\right)^2 \{\exp[\frac{-2(t^3 - t_m^3)}{3t_m^2}]\}$ | $I_m t_m = \frac{2nF\rho h}{M}\{\exp[-\frac{2}{3}]\}$ | $t_m = \sqrt[3]{\frac{2\rho^2}{\pi M^2 A N_0 k_g^2}}$ |
| 3DI | $\frac{I}{I_m} = \sqrt{\frac{1.9542}{t/t_m}}\{1 - \exp\left[1.2564\frac{t}{t_m}\right]\}$ | $I_m^2 t_m = 0.1629(nFc)^2 D_0$ | $t_m = \frac{1.2564}{N_0 \pi k}$ |
| 3DP | $\frac{I}{I_m} = \sqrt{\frac{1.2254}{t/t_m}}\{1 - \exp\left[2.3367\left(\frac{t}{t_m}\right)^2\right]\}$ | $I_m^2 t_m = 0.2598(nFc)^2 D_0$ | $t_m = \sqrt{\frac{4.6733}{A N_\infty \pi k' D_0}}$ |

ρ, M: the density and molecular weight of the $Li_2S$, respectively; $M_{Li2S}$=46 g mol$^{-1}$, $\rho_{Li2S}$= 166 g cm$^{-3}$.

$N_0$ and $AN_\infty$: the number density of deposited material, respectively.

*nF*: the molar charge transferred during electrodeposition where *F* is Faraday constant (96485 C $mol^{-1}$)

*C*: the molar concentration.

*h*: the layer thickness.

$K_g$: the nucleus lateral growth-rate constant.

**Table.** $I_m$ and $t_m$ values obtained from the *i*-*t* curve measured on each cathode substrate cell and determination of growth type.

| Cathode substrate | $i_m$ (A $g^{-1}$) | $t_m$ (s) | Growth mode |
|---|---|---|---|
| $MnCo_2O_4$/CNT | 2.03 | 570 | 3D |
| $ZnCo_2O_4$/CNT | 1.66 | 510 | 2D |
| $NiCo_2O_4$/CNT | 2.39 | 360 | 2D |
| $CuCo_2O_4$/CNT | 3.32 | 270 | 2D |
| $Co_3O_4$/CNT | 2.38 | 320 | 2D |

**Supplementary note #4**

The redox reaction rate for the conversion between $Li_2S_6$ and $Li_2S$, known as the rate-determining step, is analyzed by cyclic voltammetry. The measurements are performed by employing symmetrical electrodes of oxide-coated CNT films in an electrolyte containing $Li_2S_6$. The CV profiles for all substrates show peaks corresponding to the conversion between $Li_2S_6$ and $Li_2S$ in anodic and cathodic sweeps.[33] The polarization values for $MnCo_2O_4$, $ZnCo_2O_4$, $NiCo_2O_4$, $CuCo_2O_4$, and $Co_3O_4$ substrates are 0.19 V, 0.21 V, 0.27 V, 0.60 V, and 0.69 V, respectively. The smallest polarization for $MnCo_2O_4$ is due to the 3D granular growth of $Li_2S$ on the substrate. The granular growth is beneficial to maintaining contact with the electrolyte of the cathode during discharge, thus facilitating the discharge reaction. Other oxide substrates obtained the 2D $Li_2S$ growth. The conformal coating passivates the cathode surface, making the discharge reaction sluggish.

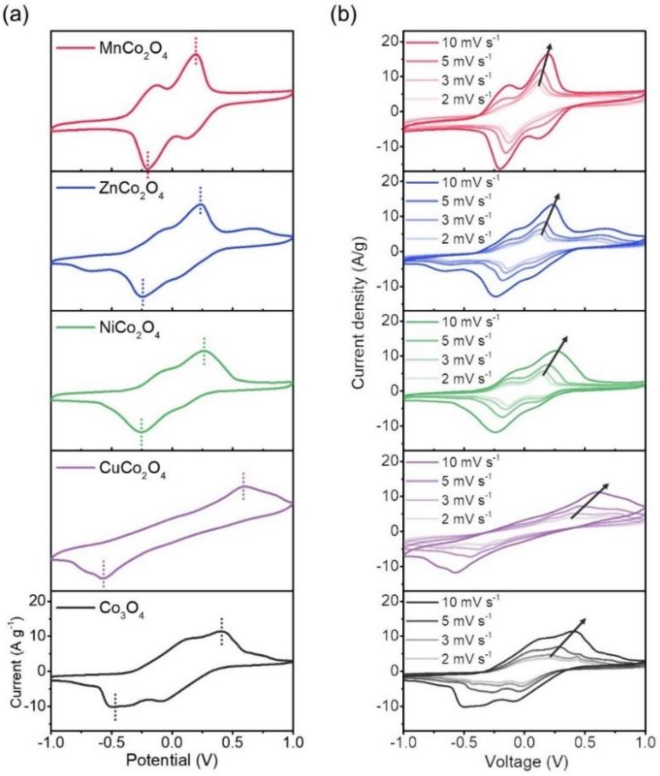


**Figure.** CV curves of (a) $MnCo_2O_4$/CNT, $ZnCo_2O_4$/CNT, $NiCo_2O_4$/CNT, $CuCo_2O_4$/CNT, and $Co_3O_4$/CNT of symmetric cells at 10 mv $s^{-1}$. (b) under the shift of scan rate, ranging from 2 – 10 mV $s^{-1}$

We also analyze the shift rate of peak potential values with increasing scan rate for each cathode substrate. This rate is related to the reaction kinetics, including the diffusion of ions on the substrate surface. The smaller the ratio, the faster the kinetics on that cathode substrate.[3] For $MnCo_2O_4$, $ZnCo_2O_4$, $NiCo_2O_4$, $CuCo_2O_4$, and $Co_3O_4$, the ratios are 105.1, 83.2, 62.1, 31.3, and 5.6 A $V^{-1}$ $g^{-1}$, respectively. (Figure 3d) The result also confirms the fastest kinetics on the $MnCo_2O_4$ substrate.

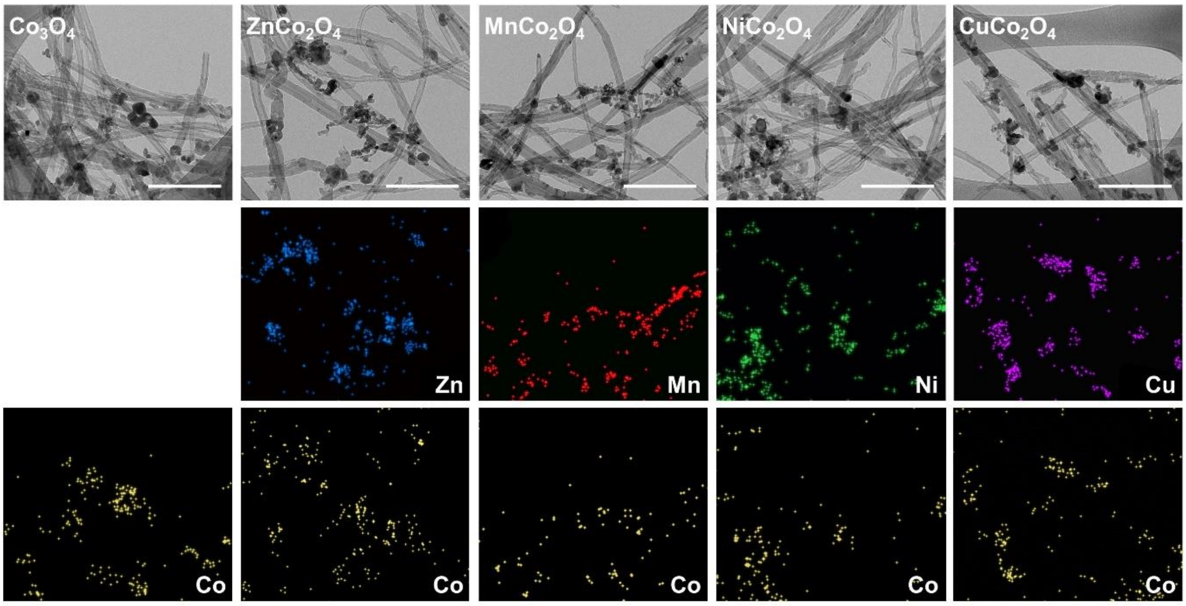


**Figure S1.** TEM and elemental mapping images of $Co_3O_4$, $ZnCo_2O_4$, $MnCo_2O_4$, $NiCo_2O_4$, and $CuCo_2O_4$ nanoparticle-coated-CNTs.

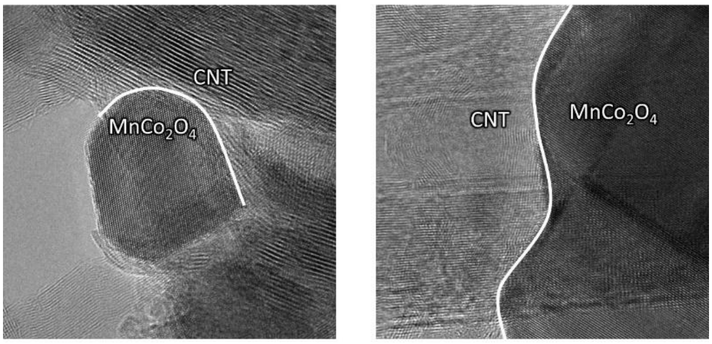


**Figure S2.** High-resolution TEM image of CNTs coated with $MnCo_2O_4$ nanoparticles. It is confirmed that the $MnCo_2O_4$ nanoparticles are bound to the CNTs with a wide interface.

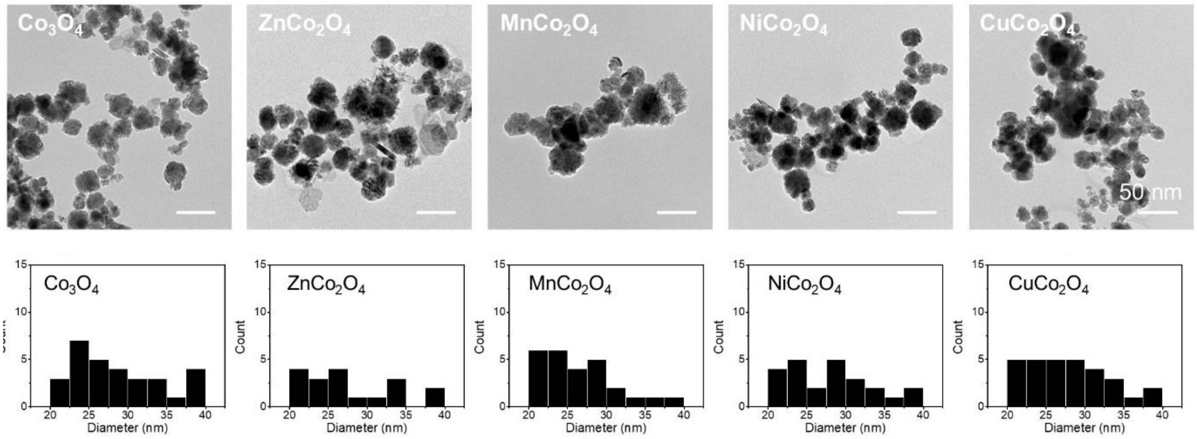


**Figure S3**. TEM images and size histograms of $Co_3O_4$, $ZnCo_2O_4$, $MnCo_2O_4$, $NiCo_2O_4$, and $CuCo_2O_4$ nanoparticles, respectively

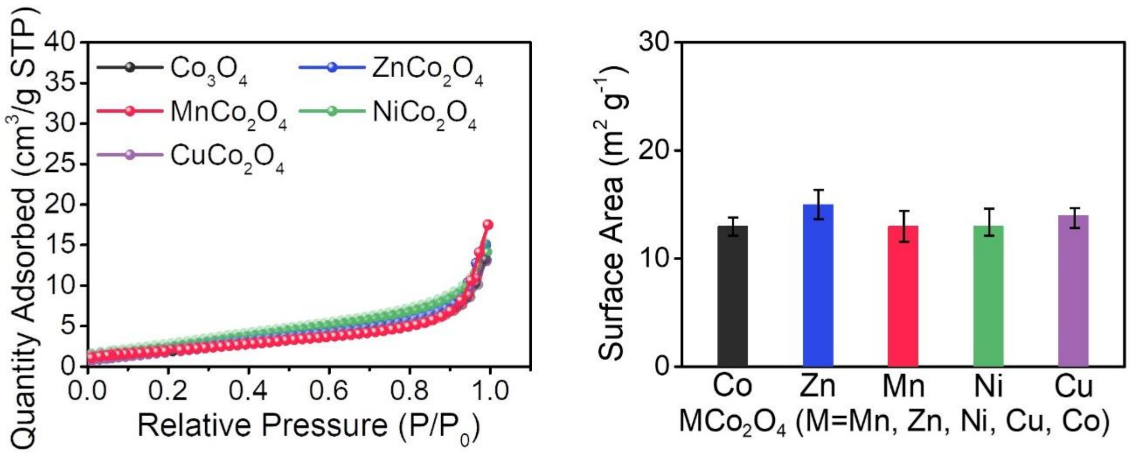


**Figure S4**. (a) BET adsorption isotherms and (b) specific areas of $MnCo_2O_4$, $ZnCo_2O_4$, $NiCo_2O_4$, $CuCo_2O_4$, or $Co_3O_4$ coated CNT film. For each sample, the mean and standard deviation of three sample runs is presented.

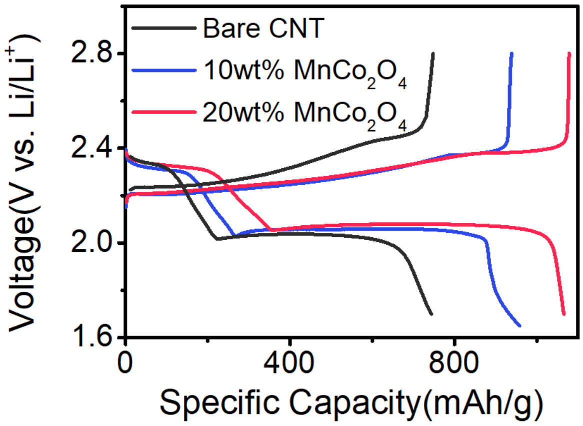


**Figure S5.** Discharge and charge profiles of $MnCo_2O_4$/CNT cells containing 10 wt % and 20 wt % of $MnCo_2O_4$. The profile of bare CNT cells is also presented for comparison. Higher cell capacity was obtained at higher $MnCo_2O_4$ content; Based on this result, the content of all oxides was controlled to be 20wt%.

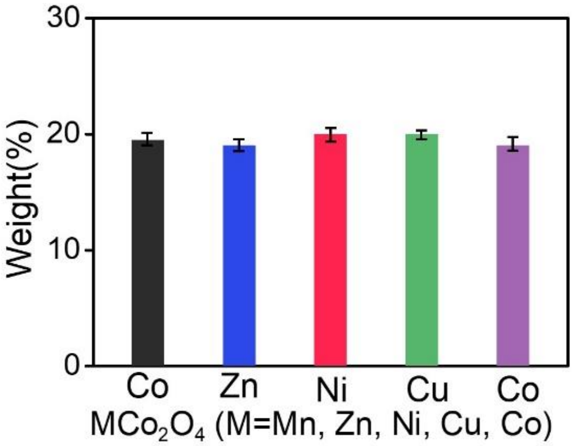


**Figure S6**. TGA results of CNTs coated with $MnCo_2O_4$, $ZnCo_2O_4$, $NiCo_2O_4$, $CuCo_2O_4$, and $Co_3O_4$, respectively. The mass content of oxides in CNTs coated with ternary oxides and $Co_3O_4$ nanoparticles is similar in the range of 18 - 20 wt%.

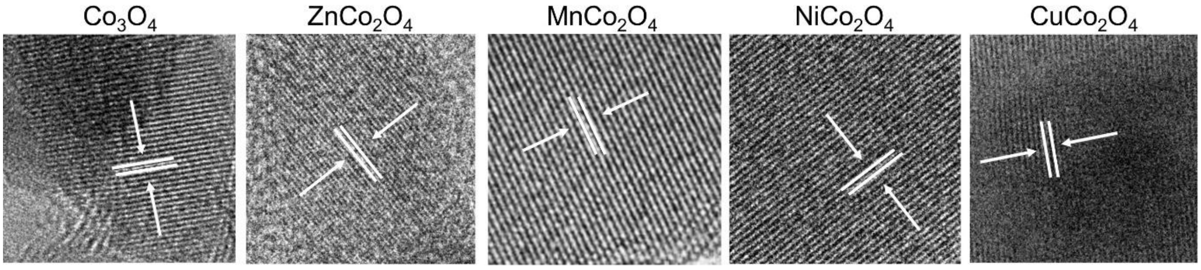


**Figure S7**. HR-TEM images of $MnCo_2O_4$, $ZnCo_2O_4$, $NiCo_2O_4$, $CuCo_2O_4$, and $Co_3O_4$ nanoparticles. The lattice parameters measured for each image are 0.24, 0.24, 0.25, 0.24, and 0.24 nm, respectively, which are consistent with the results from the literature.[34-37]

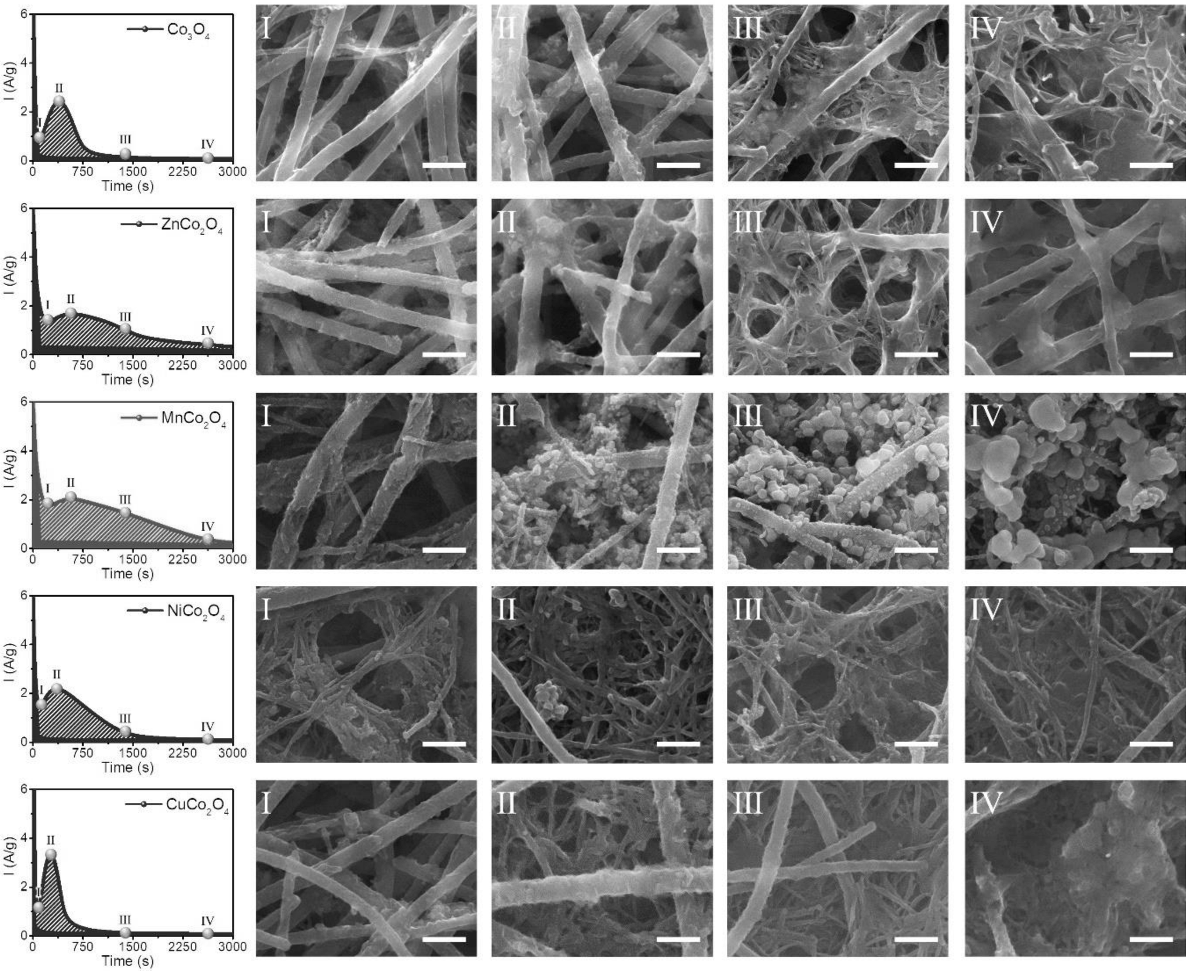


**Figure S8**. SEM images of $Co_3O_4$/CNT, $ZnCo_2O_4$/CNT, $MnCo_2O_4$/CNT, $NiCo_2O_4$/CNT, and $CuCo_2O_4$/CNT electrodes under a potentiostati discharge of 2.05 V; The SEM images show the electrode surface at points I, II, III and IV for each electrode. (scale bar: 500 nm)

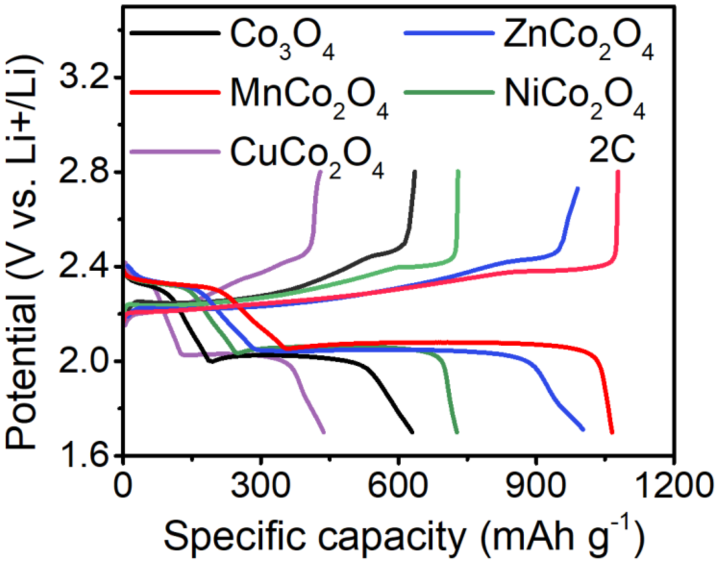


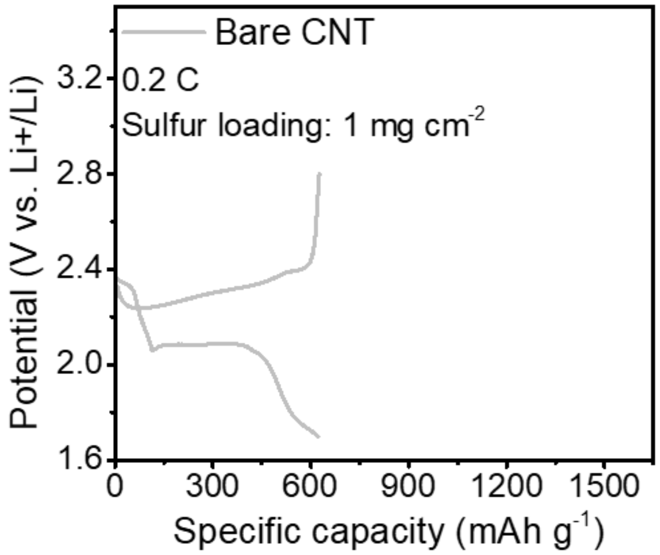


**Figure S9**. (Top) Galvanostatic discharge/charge profiles in ternary oxide/CNT cathode cells at 2 C. (Bottom) Galvanostatic discharge/charge profiles in bare CNT cathode cell at 0.2 C.

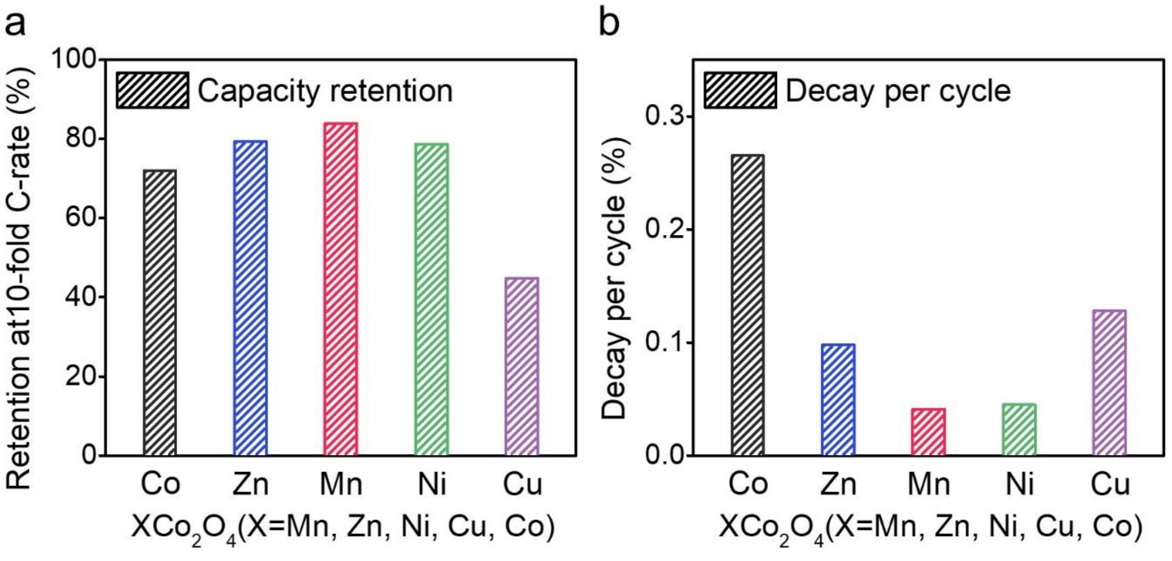


**Figure S10**. a) Capacity retention at 10-fold C-rate increase and b) capacity reduction per cycle up to 200 cycles for $MnCo_2O_4$, $ZnCo_2O_4$, $NiCo_2O_4$, $CuCo_2O_4$ or $Co_3O_4$-coated CNT cells.

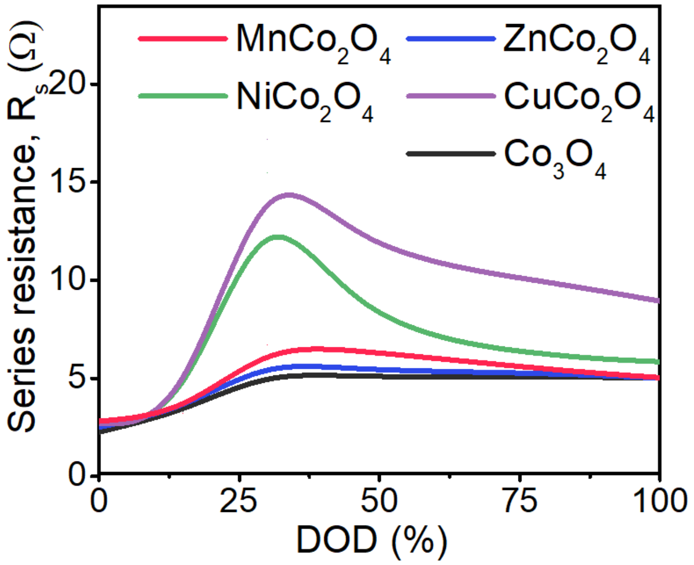


**Figure S11**. Series resistance versus depth of discharge (%) of $MnCo_2O_4$, $ZnCo_2O_4$, $NiCo_2O_4$, $CuCo_2O_4$, and $Co_3O_4$, measured by *operando* EIS technique.

**Reference**

[1] Q. Espresso, *J. Phys.: Condens. Matter* **2009**, *21*, 395502.
[2] G. Zhou, S. Zhao, T. Wang, S.-Z. Yang, B. Johannessen, H. Chen, C. Liu, Y. Ye, Y. Wu, Y. Peng, *Nano Lett.* **2019**, *20*, 1252-1261.
[3] J. Zhou, X. Liu, L. Zhu, J. Zhou, Y. Guan, L. Chen, S. Niu, J. Cai, D. Sun, Y. Zhu, *Joule* **2018**, *2*, 2681-2693.
[4] P. Qiu, Y. Yao, W. Li, Y. Sun, Z. Jiang, B. Mei, L. Gu, Q. Zhang, T. Shang, X. Yu, *Nano Lett.* **2020**, *21*, 700-708.
[5] Z. Ye, Y. Jiang, T. Yang, L. Li, F. Wu, R. Chen, *Adv. Sci.* **2022**, *9*, 2103456.
[6] Q. Liang, J. Chen, Y. Zhou, J. T. Li, L. Huang, S. G. Sun, *Adv. Funct. Mater.* **2021**, *31*, 2104513.
[7] R. Sun, Y. Bai, Z. Bai, L. Peng, M. Luo, M. Qu, Y. Gao, Z. Wang, W. Sun, K. Sun, *Adv. Energy Mater.* **2022**, *12*, 2102739.
[8] T. Sun, X. Zhao, B. Li, H. Shu, L. Luo, W. Xia, M. Chen, P. Zeng, X. Yang, P. Gao, *Adv. Funct. Mater.* **2021**, *31*, 2101285.
[9] L. Zhang, Y. Liu, Z. Zhao, P. Jiang, T. Zhang, M. Li, S. Pan, T. Tang, T. Wu, P. Liu, *ACS nano* **2020**, *14*, 8495-8507.
[10] Z. Cao, J. Guo, S. Chen, Z. Zhang, Z. Shi, Y. Yin, M. Yang, X. Wang, S. Yang, **2021**.
[11] W. Yao, C. Tian, C. Yang, J. Xu, Y. Meng, I. Manke, N. Chen, Z. Wu, L. Zhan, Y. Wang, *Adv. Mater.* **2022**, 2106370.
[12] X. Gao, Y. Huang, X. Li, H. Gao, T. Li, *Chem. Eng. J.* **2021**, *420*, 129724.
[13] Y. Wei, B. Wang, Y. Zhang, M. Zhang, Q. Wang, Y. Zhang, H. Wu, *Adv. Funct. Mater.* **2021**, *31*, 2006033.
[14] W. Wang, L. Huai, S. Wu, J. Shan, J. Zhu, Z. Liu, L. Yue, Y. Li, *ACS nano* **2021**, *15*, 11619-11633.
[15] W. Qiu, G. Li, D. Luo, Y. Zhang, Y. Zhao, G. Zhou, L. Shui, X. Wang, Z. Chen, *Adv. Sci.* **2021**, *8*, 2003400.
[16] Y. Wang, R. Zhang, J. Chen, H. Wu, S. Lu, K. Wang, H. Li, C. J. Harris, K. Xi, R. V. Kumar, *Adv. Energy Mater.* **2019**, *9*, 1900953.
[17] R. Sun, Y. Bai, M. Luo, M. Qu, Z. Wang, W. Sun, K. Sun, *ACS nano* **2020**, *15*, 739-750.
[18] W. Sun, Y. Li, S. Liu, C. Liu, X. Tan, K. Xie, *Chem. Eng. J.* **2021**, *416*, 129166.
[19] Z. Qiao, Y. Zhang, Z. Meng, Q. Xie, L. Lin, H. Zheng, B. Sa, J. Lin, L. Wang, D. L. Peng, *Adv. Funct. Mater.* **2021**, *31*, 2100970.

[20] Y. Li, Z. Li, C. Zhou, X. Liao, X. Liu, X. Hong, X. Xu, Y. Zhao, L. Mai, *Chem. Eng. J.* **2021**, *422*, 130107.

[21] Y. Li, D. Lei, T. Jiang, J. Guo, X. Deng, X. Zhang, C. Hao, F. Zhang, *Chem. Eng. J.* **2021**, *426*, 131798.

[22] D. Fang, G. Wang, S. Huang, T. C. Li, J. Yu, D. Xiong, D. Yan, X. L. Li, J. Zhang, Y. Von Lim, *Chem. Eng. J.* **2021**, *411*, 128546.

[23] C. Ma, Y. Zhang, Y. Feng, N. Wang, L. Zhou, C. Liang, L. Chen, Y. Lai, X. Ji, C. Yan, *Adv. Mater.* **2021**, *33*, 2100171.

[24] Y. Liu, W. Kou, X. Li, C. Huang, R. Shui, G. He, *Small* **2019**, *15*, 1902431.

[25] Q. Pang, C. Y. Kwok, D. Kundu, X. Liang, L. F. Nazar, *Joule* **2019**, *3*, 136-148.

[26] J. Qian, Y. Xing, Y. Yang, Y. Li, K. Yu, W. Li, T. Zhao, Y. Ye, L. Li, F. Wu, *Adv. Mater.* **2021**, *33*, 2100810.

[27] W. Weng, J. Xiao, Y. Shen, X. Liang, T. Lv, W. Xiao, *Angew. Chem. Int. Ed.* **2021**, *60*, 24905-24909.

[28] Z. Liang, D. Yang, P. Tang, C. Zhang, J. Jacas Biendicho, Y. Zhang, J. Llorca, X. Wang, J. Li, M. Heggen, *Adv. Energy Mater.* **2021**, *11*, 2003507.

[29] D. Yang, C. Zhang, J. J. Biendicho, X. Han, Z. Liang, R. Du, M. Li, J. Li, J. Arbiol, J. Llorca, *ACS nano* **2020**, *14*, 15492-15504.

[30] B. Scharifker, G. Hills, *Electrochim. Acta* **1983**, *28*, 879-889.

[31] B. Scharifker, R. Rugeles, J. Mozota, *Electrochim. Acta* **1984**, *29*, 261-266.

[32] A. Bewick, M. Fleischmann, H. Thirsk, *Transactions of the Faraday Society* **1962**, *58*, 2200-2216.

[33] G. Zhou, H. Tian, Y. Jin, X. Tao, B. Liu, R. Zhang, Z. W. Seh, D. Zhuo, Y. Liu, J. Sun, *PNAS* **2017**, *114*, 840-845.

[34] K. Jiang, B. Liu, M. Luo, S. Ning, M. Peng, Y. Zhao, Y.-R. Lu, T.-S. Chan, F. M. de Groot, Y. Tan, *Nat. Commun.* **2019**, *10*, 1-9.

[35] Y. T. Liu, D. D. Han, L. Wang, G. R. Li, S. Liu, X. P. Gao, *Adv. Energy Mater.* **2019**, *9*, 1803477.

[36] D. Wu, H. Han, X. Hong, S. Tao, S. Xu, B. Qian, L. Wang, X. Chen, P. K. Chu, *J. Alloys Compd.* **2020**, *846*, 155720.

[37] V. Mani, S. Selvaraj, T.-K. Peng, H.-Y. Lin, N. Jeromiyas, H. Ikeda, Y. Hayakawa, S. Ponnusamy, C. Muthamizhchelvan, S.-T. Huang, *ACS Applied Nano Materials* **2019**, *2*, 5049-5060.
